# Preparation and Evaluation of Zeolite Nanoparticles as a Delivery System for *Helicoverpa armigera* Nucleopolyhedrovirus (*Ha*NPV) against the *Spodoptera litura* (Fabricius, 1775) Larvae

**DOI:** 10.3390/microorganisms11040847

**Published:** 2023-03-27

**Authors:** Mia Miranti, Camellia Panatarani, I Made Joni, Maharani Herawan Ossa Putri, Hikmat Kasmara, Melanie Melanie, Desak Made Malini, Wawan Hermawan

**Affiliations:** 1 Department of Biology, Faculty of Mathematics and Natural Sciences, Universitas Padjadjaran, Sumedang 45363, West Java, Indonesia; 2 Department of Physic, Faculty of Mathematics and Natural Sciences, Universitas Padjadjaran, Sumedang 45363, West Java, Indonesia; 3 Functional Nano Powder University Center of Excellence, Universitas Padjadjaran, Sumedang 45363, West Java, Indonesia

**Keywords:** delivery system, nucleopolyhedrovirus, *Spodoptera litura* (Fabr.), zeolite nanoparticles

## Abstract

Synthetic insecticides frequently cause pest resistance and destroy non-target organisms. Thus, virus formulation is an issue that deserves considerable attention in developing virus-based insecticides. The hindrance of using nucleopolyhedrovirus alone as a virus-based insecticide is due to slow lethal time, though its mortality remains high (100%). This paper reports the formulation of zeolite nanoparticles as a delivery system to accelerate lethal time in controlling *Spodoptera litura* (Fabr.). Zeolite nanoparticles were prepared using the beads-milling method. The statistical analysis was carried out by a description exploration method with six replications. The occlusion bodies’ concentration in the virus formulation was 4 × 10^7^ OBs in 1 mL medium. Zeolite nanoparticles formulation sped up the lethal time significantly (7.67 days) compared to micro-size zeolite (12.70 days) and only nucleopolyhedrovirus (8.12 days) and received acceptable mortality (86.4%). The zeolite nanoparticles delivery system provides an alternative formulation for nucleopolyhedrovirus with a significantly improved speed of killing the virus while maintaining suitable efficacy of the virus preparation in terms of the prevalence of mortality.

## 1. Introduction

*Spodoptera litura* (Fabr.) (Lepidoptera: Noctuidae), also known as armyworm, is a polyphagous and widely distributed pest of many vegetables, fields, and ornamental crops [1,2]. The larvae are one of the major pests of many important crop plants, which attack more than 112 species of cultivated crops, including crops of economic importance [2]. Using synthetic insecticides for controlling *S. litura* may lead to the development of resistant pest populations and negatively affect non-target organisms [3]. In the past years, many researchers explored alternative control methods for insect pests, particularly *S. litura*, to reduce the use of synthetic insecticides. [1,3].

Microbial agents (viruses, bacteria, and fungi) or their bioactive agents can be used as active substances and therefore are referred to as microbial pest control agents (MPCA) [4]. They are used as alternative methods to counteract resistance and reduce adverse environmental and human health effects caused by chemical pesticides [5]. Baculoviruses are entomopathogenic viruses with a narrow host range; therefore, they are considered safe biological control agents [6]. Baculovirus consists of a double-stranded deoxyribonucleic acid genome covered with protein capsid. The lipoprotein envelope surrounds the nucleocapsid, and then, the virions are packaged in occlusion bodies called polyhedral. Occlusion bodies are composed of a matrix that occludes the virions and an outer membrane-like structure on the surface. The major protein-forming occlusion bodies are polyhedrin/granulin [7].

Viruses as biocontrol agents are an essential component of integrated pest management. This control method has been used for significant insect pests [3]. For example, baculoviruses have almost exclusively been used as insecticides for some important insect pests [8]. Additionally, nuclear polyhedrosis virus (NPV) has been commercially available as biopesticides and extensively used for controlling *Helicoverpa armigera* [7]. This commercial viral product is known as “helicovex” [9]. However, the application of baculovirus to control the population of insect pests is not widely adopted [8,10] because of its slow killing action [3,8] and fast degradation under environmental conditions, especially UV radiation [3,11]. In addition, to solve the application challenges, NPV was subcultured into the new host of *S. litura* larvae called *Helicoverpa armigera* nucleopolyhedrovirus subculture (*Ha*NPV_1_).

Zeolites are microporous, chemically inactive, crystalline aluminosilicates with promise as crop protection agents [12,13,14]. Zeolites have been widely used for odor adsorption and in agriculture for improving soil properties as a slow-release carrier of agrochemicals and as a feed additive to adsorb mycotoxins. Adjuvants, such as zeolite minerals, can be used as a carrier material (i.e., a delivery system) for viruses, and they potentially have insecticidal properties [13]. For instance, the residual application of a zeolite particle film significantly increased the mortality of eggs and larvae of the tomato leafminer, Tuta absoluta (Lepidoptera: Gelechiidae) [13]. 

The use of nanotechnology to design and manufacture targeted pesticides with an environmentally benign, controlled release through chemical modifications and compounds has been suggested to offer great potential for creating new formulations (citation). Nanopesticides for plant protection is an emerging field of research, and nanopesticide-based formulations can help the development of more effective and safer pesticides and biopesticides (e.g., microbial and naturally occurring biochemical pesticides) [15,16]. For example, a nanoparticle was used as a nanocarrier for bioactive ingredients of *Suaeda maritima*-based herbal coils as potential biopesticides against the dengue vector *Aedes aegypti* (Diptera: Culicidae) and the tobacco cutworm *Spodoptera litura* (Lepidoptera: Noctuidae) [17]. Thus, this technology might help improve management for other pests, such as *S. litura*, by increasing the effective use of pesticides and decreasing pesticide residue [18].

Nano-sized zeolites have been found to be more reactive than conventional micro-sized zeolites [12]. However, the application of nanoparticles in suspension depends on particle size, particle size distribution, and suspension stability [6,14]. Thus, this research aimed to assess zeolite nanoparticles (ZNPs) as a delivery system for HaNPV1 against S. litura larvae. Micro-sized zeolite and ZNPs suspensions were developed using a beads-milling method, and the particle size, particle size distribution, and stability of the suspension were evaluated using zeta and particle size analyzer. Moreover, the advanced measurement system was utilized to investigate the morphology of particles and viruses employing a scanning electron microscope (SEM). Finally, the newly developed ZNP was compared to micro-sized zeolite and *Ha*NPV_1_ (control) by assessing the mortality and lethal time of *S. litura* exposed to these products.

## 2. Materials and Methods

### 2.1. Culture of Spodoptera litura

Fourth instars *S. litura* larvae have been taken from the Indonesian Vegetables Research Institute, in 517 Tangkuban Parahu, Lembang, West Java, Indonesia 40,391 (latitude, longitude: −6.801655, 107.649076). These insect larvae were kept in 1000 mL plastic containers covered with insect screen cloth and reared in a growth chamber at 28.1 °C and 12:12 h (light:dark) photoperiod [19]. Insecticide-free cabbage leaves were offered as feed, and larvae were used when they reached the third instar. The growth chamber device used a Lutron HT-3006A for humidity, temperature digital meter with humidity accuracy at 25 °C was 1% R.M., and temperature accuracy was 0.8 °C, as reported in our previous study [19].

The larval were reared until the pupal stage. The pupae were placed in a rearing container with soil as pupal substrate until they hatched into the imago. The imago cage is equipped with ovitrap, which is fed with 10% honey solution. The eggs are separated in a different container until they hatched into the first instar larvae [20]. Larval instar transition is determined through the molting phase. Each instar stage is moved to a separate container. The different stages of instar larvae were determined and confirmed by the measure of morphological width of the head capsule of each larval instar with Dyar’s law formula and calculation of the average. The third instar larval was used for the experiment [2] nuclear polyhedrosis virus that has been subcultured in the new host of *S. litura* larvae called *Helicoverpa armigera* nucleopolyhedrovirus subculture (*Ha*NPV_1_)_._

Virus preparation materials using sodium dodecyl sulphate (Merck KGaA, Darmstadt, Germany), Tris buffer (Merck KGaA, Darmstadt, Germany), sodium chloride (Merck KGaA, Darmstadt, Germany), Aquadest (Bratachem, Jakarta, Indonesia), and sodium azide (Merck KGaA, Darmstadt, Germany). Natural zeolite is used as a delivered medium for virus formulation.

### 2.2. The Propagation of Nuclearpolyhedrosis Virus Suspension

Originally, *Ha*NPV was isolated from *H. armigera* larvae as the main host with a production rate of 9.5 × 10^9^ OBs mL^−1^. *Ha*NPV_1_ is a subculture of *Ha*NPV in *S. litura* as an alternate host with enhanced OBs production 1.55 × 10^11^ OBs mL^−1^ compared to *Ha*NPV produced in *H. armigera* as the main host.

The subculture of the *Ha*NPV_1_ culture was obtained from the Laboratory of Applied Microbiology Department of Biology, Faculty of Mathematics and Natural Sciences Universitas Padjadjaran. The *Ha*NPV_1_ was produced by propagating the *Ha*NPV in *S. litura* as an alternate host. The virus was isolated after one passage subculture in *S. litura* larvae. *Spodoptera litura* third instar larval were infected by a virus suspension with a concentration of 4 × 10^5^ occlusion bodies (OBs) mL^−1^. The infected larval was collected in a 100 mL glass chamber and stored at 4 °C. Then, the cadavers of 40 larvae in the same cohort were crushed by mortar and mixed with 20 mL Tris buffer solution (1 mM, pH 7.6) and 20 mL 0.1% sodium dodecyl sulfate (SDS) solution. This concentrate was stored at 4 °C for 24 h [21].

After storage, the concentration of the virus was filtered with two layers of muslin cloth in a sterilized 100 mL glass chamber. The 8 mL suspension of the virus was transferred to a 10 mL centrifuge tube with a pipette. Tubes were placed in a centrifuge, and the virus suspension was processed at 3500 rpm for 15 min at 4 °C. After 15 min of the first centrifugation, the virus suspension was divided into a supernatant solution and a solid precipitated polyhedral. Then, the solid precipitated polyhedral was washed three times with the following procedure. The supernatant solution was discarded, and the solid precipitated polyhedral was removed with a pipette to the centrifuge tube. The solid precipitated polyhedral was resuspended in 5 mL Tris buffer solution (1 mM, pH 7.6) and mixed with 5 mL 0.1% SDS solution. The mixture was centrifuged at 3500 rpm for 15 min at 4 °C. After washing three times, the solid precipitated polyhedral was resuspended into a mixture of Tris buffer solution (1 mM, pH 7.6) and 0.1% SDS solution by adding 0.2% sodium azide.

To count the numbers of the virus OBs, 0.1 mL of resuspended virus pellet was mixed by adding 0.9 mL mixed solution of Tris buffer (1 mM, pH 7.6) and 0.1% SDS with a 1:1 ratio. Counts were done with a Neubauer hemocytometer by a light microscope with a magnification of 400. The presence of OBs in the suspension was the cuboidal shape and green color. The virus structure was observed by scanning electron microscope (SEM/JEOL-JSM 6360LA) magnifier 30,000×. The concentration of stock virus suspension is 2.6 × 10^9^ OBs/mL solution. The virus suspension with 4 × 10^7^ OBs/mL concentration in the suspension medium was used for bioassay.

### 2.3. Preparation of HaNPV_1-_ZNPs Formula

Zeolite nanoparticles were prepared by the beads-milling method. Bead milling is one of the equipment or top-down processes for the size reduction of bulk particles into fine particles [22,23]. Commercial zeolite powder was selected to obtain a more uniform millimeter size (around 1–2 mm), subject to the beads-milling process. The beads milling consists of a 250 mL vessel, a pump, and a mixing tank. The vessel was filled with beads up to 70% capacity. The slurry was prepared by mixing the zeolite powder and distilled water. The mixed zeolite was stirred using a magnetic stirrer for 30 min before the beads milling. The PEG 400 dispersing agent was added after the mixing time of 15 min. The zeolite slurry was pumped into the vessel (with an optimized recirculation mass flow rate of 8 L/min), which contained zirconia beads. The impeller operated at a speed of 4070 rpm. Zirconia beads were agitated in the lower portion of the vessel (dispersing section) to break up the aggregate to avoid agglomeration of zeolites in the suspension.

After dispersion, the suspension was pumped from the dispersing section to the separation region where centrifugal force was used to separate the zirconia beads from the particle suspension. The zeolite suspension was then recycled back to the dispersing section. To keep the temperature of the system constant, the vessel was supported by a water jacket system and was completely sealed. The PEG 400 content in suspension was 150 weight fraction of zeolites. The water-suspended ZNPs with weight fractions of 0.1 wt%, milling time of 120 min, and addition of acetic acid solution (1 vol%) after milling to promote the positive surface change were used for optimal technical conditions for beads milling.

### 2.4. Characterizations

The particle size distribution of ZNPs was measured using dynamic light-scattering (DLS) equipment with A DelsaTM nano series instrument (Beckman Coulter, Inc., Brea, CA, USA). Micro-sized zeolite powder was mixed in water, and the concentration of ZNPs in the suspension was 1%.

### 2.5. Formulation of HaNPV_1_-ZNPs

Virus formulation with zeolite (Z) as an adjuvant was made by mixing 15 mL stock virus solution and 1000 g zeolite. The mixture was stirred evenly and dried at 22–28 °C for a week. Virus formulation in ZNPs (*Ha*NPV_1_: ZNPs) as an adjuvant was made by mixing 15 mL stock virus solution and 1000 mL ZNPs suspension. Virus formulation in Z and ZNPs can be used for larval infection. The virus suspension concentration formulated in Z and ZNPs was 4 × 10^7^ OBs gram powder or mL^−1^ solution. Both Z and ZNPs suspension as an adjuvant without virus were used for bioassay test larvae.

### 2.6. The Bioassay Tests

The third instar larvae of *S. litura* were used for a bioassay test and replicated six times. Virus formulation of 0.30-g Z or 0.30 mL ZNPs suspension and in-suspension medium were spread on 10 cm diameter of free synthetic insecticides cabbage leaves. The infection process was conducted by inserting five larvae into the container provided with treated leaves. The larvae were starved for 3 h before treatment. After 24 h of infection, the feed was replaced daily with fresh cabbage leaves. Larval mortality was recorded for 14 days. Mortality (%) was defined as the number of infected larvae that died out of the total number of larvae tested during observation. The lethal time was defined as the time required for larvae to die after a virus infection.

### 2.7. Data Analysis

The total number of polyhedral was calculated using the formula as follows:

(1) $$\frac{N}{V}\times P\times 10^{3}$$

where *N* is the number of polyhedral counted, *V* is the area counted (Neubauer counting chamber), and *P* is the dilution factor.

The average lethal time was calculated using the formula in Equation (1) for 14 days of observation, according to Prasetio et al. [24] as follows.

(2) $$W=\frac{\sum W_{i}\times Z_{i}}{Y}$$

*W* is the average lethal time, *W_i_* is the lethal time on day *i* (and not day 1), *Z_i_* is the number of insects that died on day *i* (and not day 1), and *Y* is the total number of insects that died. The data were collected and organized using Microsoft Excel (Version 16, Microsoft Corporation, Redmond, WA, USA). Data distribution showed homogeneity. The obtained data were analyzed by ANOVA using IBM SPSS Statistics (version 22). Their significance was determined by Duncan’s multiple distance test (DMRT) with *p*-values < 0.05 considered significant.

## 3. Results and Discussion

### 3.1. The Morphology of the Occlusion Bodies of HaNPV_1_

**Figure 1:**
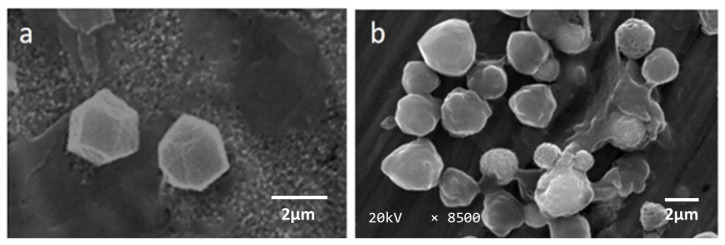
Morphological structure of (**a**) *Helicoverpa armigera* nuclear polyhedrosis virus (*Ha*NPV) and (**b**) *Helicoverpa armigera* nuclear polyhedrosis virus subcultured (*Ha*NPV_1_) occlusion bodies/OBs or polyhedra isolated from *Spodoptera litura* (Fabr.) larvae.

Figure 1 shows the SEM images of the morphology comparison of occlusion bodies (OBs) of *Ha*NPV and *Ha*NPV_1_. The morphology of *Ha*NPV OBs, as shown in Figure 1a, was a hexagonal shape, which is the usual structure of OBs when they propagated in the main host. In contrast, the morphology of *Ha*NPV_1_ OBs in an alternate host showed a spherical shape and porous structure. SEM images observed the subculture polyhedral’s spherical and porous structure shape with magnification of 8500 (Figure 1b). The modification of the structure of *Ha*NPV polyhedral in an alternate host was reported in our previous study [24]. A distinguishing feature of baculoviral is the presence of OBs, which are critical for the transmission of the virus in host insects and the survival of the virus outside the host [25]. In the present study, the OBs of the virus isolate exhibited irregular shapes, a typical characteristic of OBs of NPV belonging to alpha baculovirus [26].

### 3.2. SEM and EDS of the ZNPs

**Figure 2:**
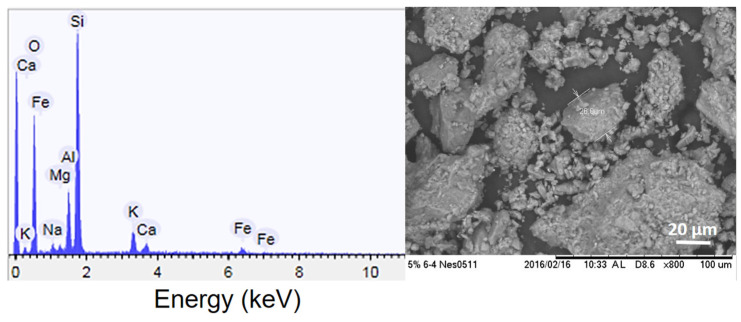
SEM Image and EDS Analysis of Raw Zeolite Particle.

Figure 2 shows the SEM images and EDS of the Z as raw material. SEM images indicated that they contained large-size powder (~200 µm) and irregular morphology. EDS spectrum showed that Z contained the chemical composition of minerals, such as Si, Al, and O, to form silica (SiO_2_), alumina (Al_2_O_3_), or aluminosilicate (AlSi). This Z also contained alkaline earth metals (Na, K, Ca) and other elements, such as Mg, Na, and Fe.

### 3.3. Size Distribution of ZNPs Suspension

**Figure 3:**
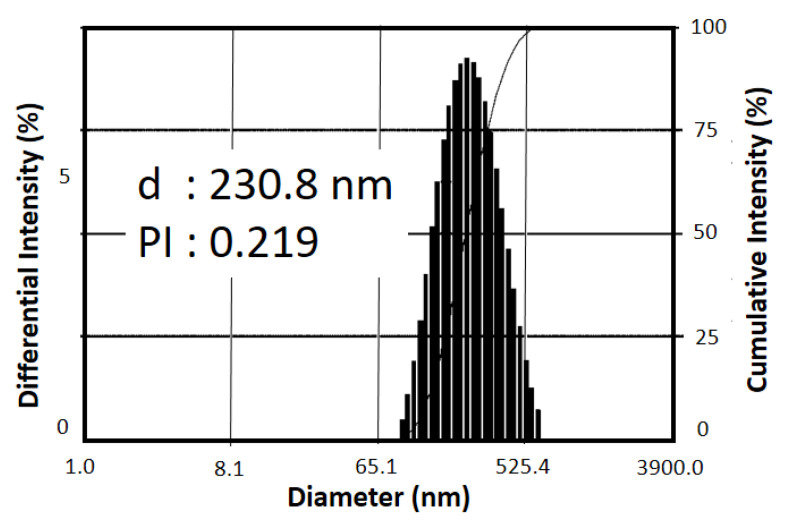
Particle Size Analysis of ZNPs Suspension.

Figure 3 shows the size distributions of the prepared ZNPs suspension by bead milling. The average size of ZNPs after the beads-milling process is approximately 230.8 nm. This result indicated that the use of PEG 400 and the addition of acetic acid solution successfully promoted a well-dispersed suspension as indicated by the monodispersed distribution of particles. Particle size is a crucial factor in determining insecticidal performance.

### 3.4. Mortality of S. litura against HaNPV_1_-ZNPs

In this study, larvae infected by *Ha*NPV_1_ suspension were used as the control and resulted in 100% mortality. The mortality of larvae exposed to Z was 37%, while ZNPs caused 49% mortality. Larval mortality of 78.16% was observed for larvae treated with *Ha*NPV_1_ + Z and of 86.40% for *Ha*NPV_1_ + ZNPs. In this condition, the existence of ZNPs as an adjuvant in the formulation causes a decrease in the viability of the virus. However, the obtained larval mortality still meets the minimum standard of efficacy of the national pesticides commission [27].

**Figure 4:**
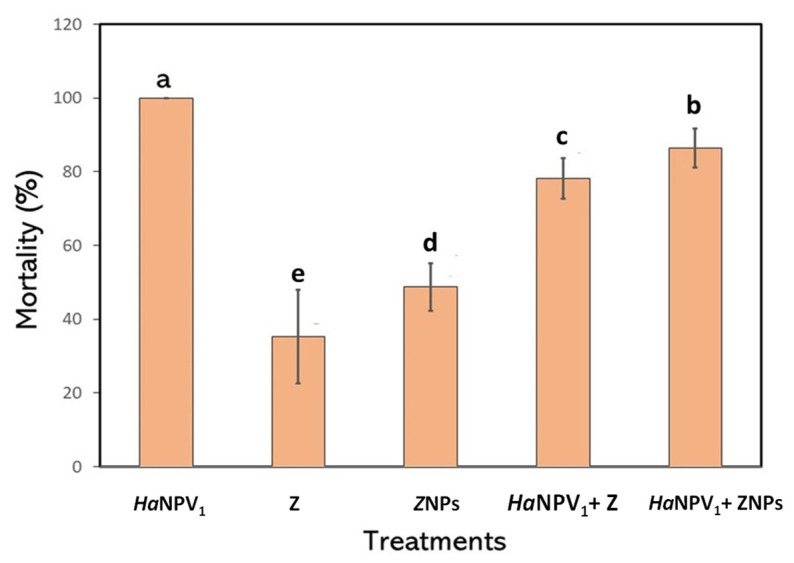
Mortality of larvae treated with different virus formulations and only the virus *Ha*NPV_1_ as a control. The letter a–e indicate significant difference within treatments (Duncan’s Multiple Range Test, *p* < 0.05).

Many researchers reported the use of ZNPs in virus formulation showed higher mortality compared to Z [12,28,29]. The smaller size of ZNPs increased their insecticidal effects due to enhancing the potential toxicity to the cells. Wijayaratne et al. (2018) reported that ZNPs were used more efficiently to penetrate infected cells than Z [28]. When a virus was used in combination with ZNPs, it showed higher mortality compared to only Z (Figure 4). The decrease in zeolite size improved the mortality of the larvae, indicating that ZNPs functioned as an adjuvant.

**Figure 5:**
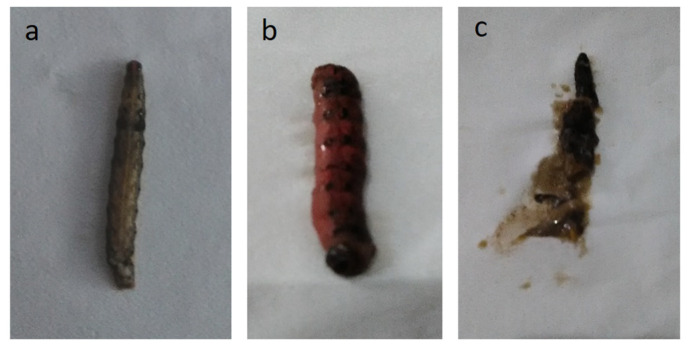
Larvae cadaver profile, (**a**) non-infected *Spodoptera litura*, (**b**) *Spodoptera litura* infected by *Helicoverpa armigera* nuclear polyhedrosis virus subcultured (*Ha*NPV_1_), and (**c**) infected larval flaccid with blackish color and stinky.

The profile of *S. litura* larval cadaver non-infected and infected by *Ha*NPV_1_ is shown in Figure 5. The symptoms of the larvae infected by the virus only and *Ha*NPV_1_ + ZNPs showed liquified bodies, abnormal body size, and stinky differences with noninfected larvae (Figure 5a). The effect of nuclear polyhedrosis virus infections against larval showed the color changes from pale to brown or blackish [30]. The bodies of larvae infected with viruses are often flaccid (Figure 5b). Figure 5c shows that the infected larvae’s integument is ruptured, as reported elsewhere [3].

### 3.5. Lethal Time of S. litura (Fabr.) against HaNPV_1_-ZNPs

**Figure 6:**
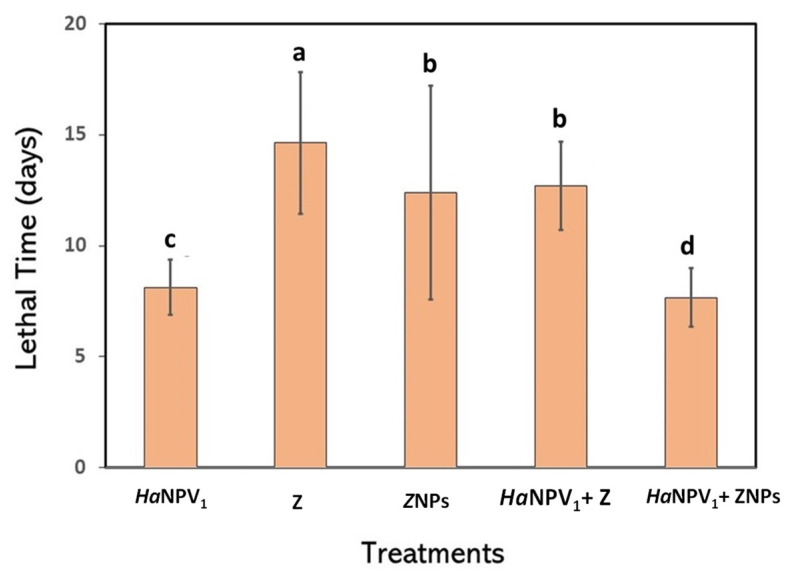
Lethal time of *Spodoptera litura* (Fabr.) infected by different formulation. The letter a–d indicate significant difference within treatments (Duncan’s Multiple Range Test, *p* < 0.05).

Figure 6 shows the average lethal time of S. litura exposed to Z, ZNPs, virus suspension, *Ha*NPV_1_ + Z, and *Ha*NPV_1_ + ZNPs. All the formulations caused a decrease in the lethal time of the infected larvae. Larvae infected only with Z and ZNPs showed lethal time of 14.6 and 12.4 days, respectively. Zeolite is effective against insects because it can abrade or adsorb the epicuticular lipids, causing rapid water loss and resulting in death by desiccation [31]. Abrasion of epicuticular lipids is attributed to sportive particles [13]. Decreasing the zeolite particles size to nano-sized zeolites accelerates larval lethal time by 20.5% than those infected by Z. This may be due to the nano-sized zeolite easily penetrating into the cells’ membrane of the larvae, which is 25 µm wide and 50 µm long [32]. The size of the virus polyhedral is 1.0–1.5 µm, covering the virus’s envelope membrane, which is 5–15 nm [33]. In addition, ZNPs provide a larger surface area than microparticles, causing higher material toxicity to the cell’s membrane. Therefore, the breakage of the cell membrane decayed cell function and caused the mortality of the larvae.

*Ha*NPV_1_ formulation in Z and ZNPs resulted in lethal times of 7.7 and 6.2 days, respectively. This indicated that ZNPs give significant acceleration at the lethal time [34]. Therefore, this result is important to resolve a critical issue where baculovirus usually needs a longer lethal time, as indicated in the control. Wider use of baculovirus as a commercial insecticide was restricted because of their slow killing action. Slow lethal time is possible for the larvae to continue feeding activity and to damage the host plant.

**Figure 7:**
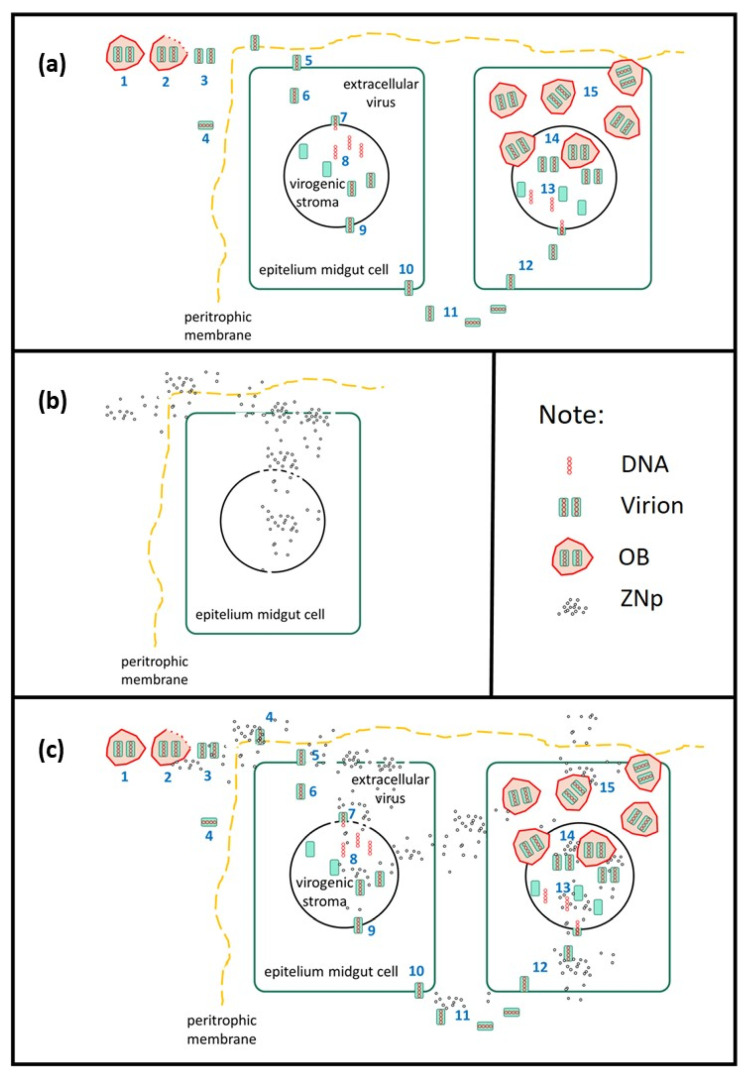
The proposed action mechanism of biocontrol to target on the lethal time: (**a**) *Ha*NPV_1_, (**b**) ZNPs, (**c**) the synergetic effect of *Ha*NPV_1_+ ZNPs. (**1.** OBs, **2**. Dissolve polyhedrin, **3**. Virions, **4**. Virions penetrate into the peritrophic membrane, **5**. Virions attached the cell membrane, **6**. Virion in the cell cytoplasm, **7**. The viral envelope attached out of the nucleus membrane, **8**. The viral genome inside the nucleus, **9**. Budded virus formation, **10**. Budded virus leaves the cell, **11**. Budded virus out of the cell, **12**. Budded virus infected the other cell, **13**. Virogenic stroma, **14**. OBs in the nucleus, **15**. OBs in the cytoplasm).

Figure 7 shows the proposed mechanism of the combined mechanism of *Ha*NPV_1_ on decreasing the lethal time when the additional ZNPs are included in the formulation. When only virus *Ha*NPV_1_ was applied, the mechanism is shown in Figure 7a. The virion attached to the membrane cell, entered the nucleus, and then released its genome. Furthermore, the virus replication occurred to form the budded virus and exited the cell to infect other cells. After infecting another cell, the virus propagated to form OBs. The significant number of OBs produced in the cell caused cell lysis. This mechanism needs longer lethal times to kill larvae. As clearly observed in Figure 6, the lethal time of only *Ha*NPV_1_ is slower compared to *Ha*NPV_1_ + ZNPs. In contrast, *Ha*NPV_1_ provides high mortality, as shown in Figure 4. On the other hand, when only ZNPs were applied, the ZNPs directly absorbed or were abrasive to the cell membrane and caused cell ruptured (Figure 7b). However, ZNPs killed larvae slower than only using *Ha*NPV_1_, as shown in Figure 6.

In contrast, when both virus (*Ha*NPV_1_) and ZNPs were applied, the synergetic mechanism occurred, as shown in Figure 7c, and was supported by lethal time and mortality observation (Figure 4 and Figure 6). The *Ha*NPV_1_ + ZNPs biocontrol agent provided faster lethal time and possessed high mortality compared to only *Ha*NPV_1_ or ZNPs. The ZNPs have a strong affinity to target such proteins and initiate the abrading to membrane cells, causing the virus to easily enter the cell [12,24]. Therefore, ZNPs with *Ha*NPV_1_ reduce the lethal time of larvae due to effectively damaging cell membranes and creating fast viral replication in midgut cells [35]. Speed of action remains an important factor in selecting strains because faster-acting strains would reduce crop damage [6,26,27].To speed up the lethal time of insect targets, it is also expected to see synergistic combinations of baculovirus with semiochemicals [36]. Thus, determining the time to kill the insect host is crucial in the case of baculovirus application.

Therefore, nanotechnology applications, such as pathogen control, have potential in crop protection [8] due to the faster-killing action and protection degradation under UV radiation. However, further studies are needed to investigate these effects under actual field conditions.

## 4. Conclusions

The larvae mortality was 100% when infected only by virus formulation (*Ha*NPV_1_) with a lethal time of up to 8.12 days. The mortality of larva against virus and micro size zeolite (*Ha*NPV_1_ + Z) was very low (80%) with a longer lethal time of up to 14.6 days. In contrast, the additional ZNPs included in the virus formulation (*Ha*NPV_1_ + ZNPs) caused 86.40% mortality and reduced the lethal time to 6.2 days. We conclude that ZNPs acted as an adjuvant and decreased the lethal time of *S. litura* significantly when infected by HaNPV1 with acceptable mortality. Thus, based on the mortality and lethal time, the best formulation was *Ha*NPV_1_ + ZNPS. Therefore, the proposed formulation has the potential to be used as an alternative to synthetic insecticide to address the critical issue of the slow lethal time of the application of *Ha*NPV_1_.

## Data Availability

Data sharing is not applicable to this article.
